# Keratin 8 variants are infrequent in patients with alcohol-related liver cirrhosis and do not associate with development of hepatocellular carcinoma

**DOI:** 10.1186/1471-230X-12-147

**Published:** 2012-10-18

**Authors:** Valentyn Usachov, Pierre Nahon, Mariia Lunova, Marianne Ziol, Pierre Rufat, Angela Sutton, Michel Beaugrand, Pavel Strnad

**Affiliations:** 1 Department of Internal Medicine I, University Hospital Ulm, Ulm, Germany; 2 Department of Hepatology, AP-HP, Jean Verdier Hospital, Bondy, F-93140, France; 3 University Paris 13, Sorbonne Paris Cité, UFR SMBH, F-93000, Bobigny, France; 4 Centre de ressources biologiques - Jean Verdier Liver Biobank-GH Paris-Seine-Saint-Denis, APHP, Bondy and Université Paris 13, Sorbonne Paris Cité, UFR SMBH, Bobigny, FRANCE; 5 Biostatistics unit, Pitié-Salpêtrière Hospital, AP-HP, Paris, France; 6 Department of Biochemistry, AP-HP, Jean Verdier Hospital, F-93140, Bondy, France; 7 Department of Internal Medicine III and IZKF, RWTH-University Hospital Aachen, Pauwelsstraße 30, D-52074, Aachen, Germany

**Keywords:** Hepatocellular carcinoma, R341H, Mortality

## Abstract

**Background:**

Keratins 8/18 (K8/K18) are established hepatoprotective proteins and K8/K18 variants predispose to development and adverse outcome of multiple liver disorders. The importance of K8/K18 in alcoholic liver disease as well as in established cirrhosis remains unknown.

**Methods:**

We analyzed the K8 mutational hot-spots in 261 prospectively followed-up patients with alcoholic cirrhosis (mean follow-up 65 months). PCR-amplified samples were pre-screened by denaturing high-performance liquid chromatography and conspicuous samples were sequenced.

**Results:**

67 patients developed hepatocellular carcinoma (HCC) and 133 died. Fourteen patients harbored amino-acid-altering K8 variants (5xG62C, 8xR341H). The presence of K8 variants did not associate with development of HCC (log-rank=0.5) or death (log-rank=0.7) and no significant associations were obtained for the single K8 variants after a correction for multiple testing was performed.

**Conclusions:**

Keratin variants are expressed in a low percentage of patients with alcoholic cirrhosis and do not influence HCC development. Further studies conducted in larger prospective cohorts are needed to find out whether presence of K8 R341H variant predispose to non-HCC-related liver mortality.

## Background

Alcohol consumption induces a variety of hepatic changes ranging from asymptomatic fatty liver to end-stage liver failure and represents the leading cause of liver-related mortality in western countries [1]. Although the pathogenesis of alcoholic liver disease (ALD) is incompletely understood, genetic factors play a significant role [2]. While most studies focused on genes involved in oxidative stress response, hepatic lipid storage, alcohol consumption/metabolism as well as liver inflammation and fibrosis [2], cytoskeletal proteins are also established ALD targets [3]. Among them, keratins represent attractive candidates to serve as modifier genes in ALD, given that (i) keratins are established liver disease modifiers [4-6]; (ii) ALD leads to a characteristic redistribution of K8/K18 network with formation of Mallory-Denk bodies and keratin redistribution was previously shown to be affected by presence of keratin variants [7,8]; (iii) oxidative stress is an integral part of ALD and presence of keratin variants primes transgenic animals towards oxidative stress [9].

Keratins (Ks) constitute the largest subgroup of intermediate filaments and are expressed predominantly in epithelial tissues as well as epithelial appendages [10,11]. They can be further subdivided into type I (K9-K28) and type II proteins (K1-K8 and K71-K80), which are both needed to form obligate heteropolymers [10,12]. Accordingly, each epithelial cell expresses at least one type I-type II keratin pair [12]. Adult hepatocytes are unique in that they produce only K8/K18 while other cells typically exhibit a more complex keratin expression pattern [12]. The simple keratin composition is likely responsible for the fact that liver is particularly sensitive to the perturbations in keratin system. To that end, multiple transgenic mice lacking K8/K18 or expressing mutated proteins are susceptible to a variety of different liver stresses [4]. Human association studies further support these data by demonstrating that K8/K18 variants predispose to development and severe outcome of various liver disorders [4-6]. K8/K18 variants are detected in 4-5% of Caucasian controls with K8 G62C/R341H being the most common ones reaching a frequency of 1.5 and 3.2%, respectively [6,13]. K8/K18 variants are not only overrepresented in subjects with multiple liver disorders, but also predispose to liver fibrosis development in patients with chronic hepatitis C and primary biliary cirrhosis as well as to adverse outcome of acute liver failure [4-6]. While these data are intriguing, it remains unclear, to what extent K8/K18 variants influence the prognosis of subjects with ALD, particularly those with already established liver cirrhosis.

Although K8/K18 are widely established hepatoprotective proteins [4], their role is likely restricted to specific liver stresses. For example, K18 R90C variant increases the susceptibility of transgenic animals to Fas-, but not TNF*a*-mediated liver injury as well as to thioacetamide-, but not carbon tetrachloride-induced liver fibrosis development [4,14]. Moreover, exonic K8/K18 variants do not significantly modulate liver fibrosis development in patients with hereditary hemochromatosis [15]. To further delineate the importance of K8/K18 variants in specific human liver disorders, we studied the impact of these variants on overall and liver-related mortality as well as on development of hepatocellular carcinoma (HCC) in a prospectively monitored cohort of French patients with alcohol-related liver cirrhosis.

## Methods

### Selection of patients

The present work was part of a prospective study conducted in cirrhotic patients that was aimed at assessing the performance of HCC screening procedures as well as the rates and risks factors of liver cancer development in the course of various liver diseases [16]. The protocol obtained approval from the Ethics Committee (CPP, Aulnay-sous-Bois, France). All patients gave written informed consent to participate in the study and all research carried out in participants was in compliance with the 1975 Declaration of Helsinki as reflected in a priori approval by the institution's human research committee.

In the present study, we included all new patients referred to the liver unit of Hôpital Jean Verdier (Bondy, France) between 1 January 1995 and 31 December 2005, who fulfilled the following criteria: 1) biopsy-proven liver cirrhosis; 2) daily alcohol intake >80 g per day 3) no infection from the human immunodeficiency virus or hepatitis B or C viruses; 4) no evidence of HCC at the time of inclusion as judged by negative ultrasonographic findings and a serum *a*-fetoprotein (AFP) level of <50 ng/mL; 5) Caucasian origin and residence in France; 6) acceptance of a regular follow-up and periodical HCC screening. A total of 261 patients were recruited.

The study started with the detection of liver cirrhosis on the first liver biopsy and all patients were prospectively evaluated at least twice a year by a physical examination, liver ultrasound and measurement of serum AFP levels. When a HCC was suspected, computer tomography, and/or magnetic-resonance imaging and/or a guided liver biopsy was performed according to the Barcelona criteria [17]. HCC was diagnosed when at least one of the following criteria were fulfilled:

1. Histological evidence

2. Congruent demonstration of a focal lesion >2 cm in size, and arterial hypervascularization, as assessed by two different imaging techniques

3. A combination of one imaging technique plus a serum AFP level of ≥400 ng/mL.

Gender, age, BMI, past history of diabetes mellitus, presence of ascites or hepatic encephalopathy, serum bilirubin, albumin and prothrombin levels, serum alanine-aminotransferase activity, and serum aspartate-aminotransferase activity were recorded at the time of inclusion. Daily alcohol intake was obtained by personal interview. The two main end-points were the occurrence of HCC and the occurrence of death or liver transplantation (the date of liver transplantation was considered as the day of death). Otherwise, the follow-up ended at the last recorded visit (or information taken) prior to 31 August 2011. This deadline was used to upgrade the patients’ file using our computerized database, as well as to obtain information by contacting the patients, their relatives or general practitioner.

### Genetic analysis

Genomic DNA was isolated from peripheral blood mononuclear cells using a commercially available kit according to manufacturer’s instructions (Amersham, GE Healthcare). Exons 1 and 6 of keratin 8 were polymerase chain reaction-amplified using established primers [18] and a “hot-start” Amplitaq Gold DNA Polymerase (Applied biosystems; Foster City, CA). The specificity of the amplification was confirmed by sequencing. PCR products were analyzed for presence of heterozygous variants by denaturing high-performance liquid chromatography (DHPLC) using a WAVE DNA Fragment Analysis system. Samples with “shifted” elution pattern were purified with a Qiaquick PCR purification kit (Qiagen) and sequenced.

### Statistical methods

Qualitative variables were compared using Fisher’s exact test, the chi-squared test or the chi-squared trend test with 1° of freedom, whereas quantitative variables were compared using the non-parametric Kruskal–Wallis test. The Kaplan–Meier method was used to estimate the occurrence of HCC/death for each parameter noted at enrolment. The distribution of death and HCC were compared with the log-rank test. A significant level <0.10 was used to select the variables for the Cox’s proportional hazards model, using a stepwise backward procedure with a threshold of *a*=0.05. Variables associated with risk of death or HCC, based on knowledge and findings from previous studies were also selected for multivariate analyses (age, gender, BMI, diabetes, Child-Pugh score, platelet count). Statistical analyses were performed using the SAS System Package version 8.02 (SAS Institute, Cary, NC). All reported *P* values are two-tailed. Associations were considered statistically significant at a *a* of 0.05.

## Results

### Patients’ characteristics

A total of 261 patients were enrolled in this study. Their initial characteristics are summarized in Table 1. The mean follow-up period was 65 months with extremes ranging from 22 to 150 months. During the study period, 24/261 patients (9.2%) were lost to follow-up after at least 2 years of HCC screening. During follow-up, 67 developed HCC and 133 died or underwent liver transplantation (*n*=17). In all but 5 cases, death was attributable to liver disease caused by advanced HCC, variceal bleeding and/or complications caused by hepatic failure. The non-HCC-related complications were the main causes of death.

**Table 1:** Characteristics of the cohort of patients with alcoholic liver cirrhosis

	**Patients with alcoholic cirrhosis**
	**(n=261)**
**Age (years)**^**a**^	56.8±0.6
**Male gender**^**b**^	202 (77.4)
**Child-Pugh score**^**a**^	7.7±0.1
**Prothrombin activity (%)**^**a**^	62.5±1.2
**Bilirubin (μmol/L) **^**a**^	47.6±3.6
**Albumin (g/L) **^**a**^	35.8±0.3
**Platelet count (10**^**3**^**/mm**^**3**^**) **^**a**^	137.2±3.2
**AST (ULN) **^**a**^	2.2±0.08
**ALT (ULN) **^**a**^	1.5±0.1
**GGT (ULN) **^**a**^	6.4±0.4
**Follow-up (months) **^**a**^	65.8±2.4
**HCC **^**b**^	67 (25.6)
**Death/liver transplant **^**b**^	133 (50.9)
HCC-related	54
Liver-related	74
Extra-hepatic	5

*ALT*, alanine aminotransferase, *AST*, aspartate aminotransferase, *GGT*, gamma-glutamyl transferase, *HCC*, hepatocellular carcinoma, *ULN*, upper limit of normal.

^a^Mean ± SEM (standard error of the mean).

^b^Number (percentage) of patients.

### Lack of influence of keratin variants on outcomes

**Figure 1:**
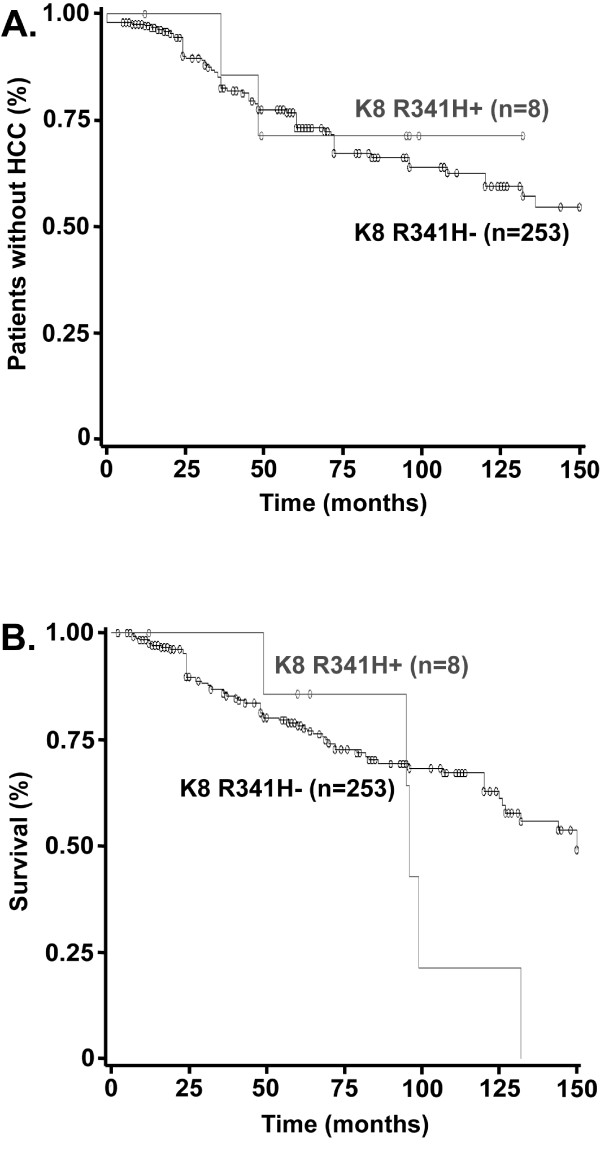
**The significance of K8 R341H variant in patients with alcoholic liver cirrhosis.** The impact of K8 R341H variant on development of hepatocellular carcinoma (HCC) (**A**) and on non-HCC-related liver mortality (**B**) was studied in subjects with alcoholic liver cirrhosis using the Kaplan–Meier method. While K8 R341H variant was not associated with HCC development (log-rank =0.7), patients carrying K8 R341H variant displayed a trend towards increased end-stage liver disease mortality (HR=2.0 [0.8-5.0], log-rank=0.1).

Our genetic analysis detected K8 G62C and K8 R341H variants in 5 and 8 patients respectively. K8 I63V variant was seen in one person and no intronic variants were observed (Table 2). When stratifying the population according to the presence of variants in K8 exon 1 (K8 G62C/I63V), exon 6 (K8 R341H) or all keratin variants (n=14), the baseline severity of liver disease as well as demographic and clinical data were similar among the variant carriers and non-carriers (data not shown). Similar rates of complications during follow-up were observed between patients bearing the K8E1 variants and the remains subjects [HCC occurrence 2/6 (33.3%) vs. 65/255 (25.5%), *P*=0.6; death: 2/6 (33.3%) vs. 126/255 (49.4%), *P*=0.4). This observation was confirmed by the Kaplan-Meier method as the K8E1 mutation carriage was not associated with the development of HCC (HR=0.8 [0.1–3.3], log-rank=0.7) or death (HR=0.4 [0.1–1.6], log-rank=0.2) when considering the occurrence of these events over time. Similarly, the presence of the K8 R341H variant was not associated with HCC occurrence [2/8 (25.0%) vs. 65/253 (25.7%), *P*=0.9], a finding that was confirmed by the Kaplan-Meier method (HR= 0.7 [0.1-3.1], log-rank =0.7, Figure 1A). On the other hand, patients carrying K8 R341H variant had an apparent higher rate of overall mortality [7/8 (87.5%) vs. 121/253 (47.8%), *P*=0.02] that was due to higher incidence of non-HCC liver-related death [5/8 (62.5%) vs. 68/253 (26.9%), *P*=0.02]. However, this negative impact was not observed when taking into account the occurrence of death over time, whether for overall mortality (HR=1.5 [0.7-3.3], log-rank =0.2) or death non-attributable to HCC (HR=2.0 [0.8-5.0], log-rank =0.1, Figure 1B).

**Table 2:** Distribution of gene mutation in the ALC patients

**Gene**	**Exon**	**Nucleotide Variants (DNA)**	**Amino Acid Variants**	**Patients**	**%**
K8	E1	184 G→T	G62C	5	1,9
		187 A→G	I63V	1*	0,38
	E6	1022 G→A	R341H	8	3,1
Total				14 (13)*	5,4 (5,0)

*Based on previous studies, K8 I63V represents a polymorphism rather than a biologically relevant K8 variant.

**Figure 2:**
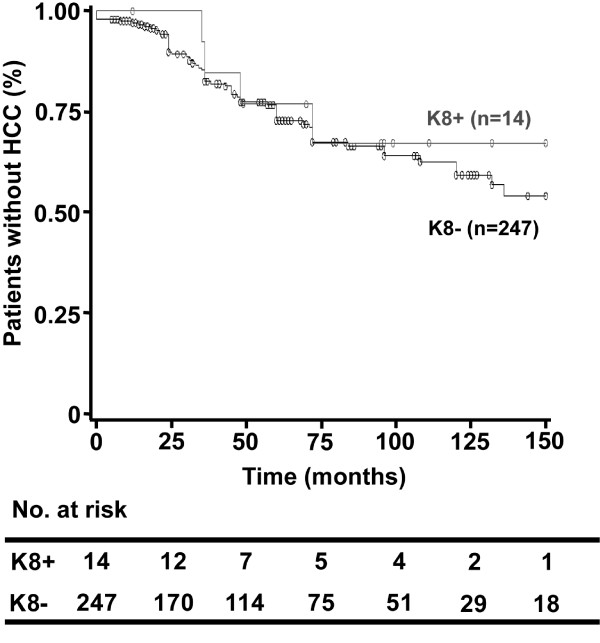
**Presence of K8 variants does not predispose to development of hepatocellular carcinoma in subjects with alcoholic liver cirrhosis.** Kaplan–Meier method was used to evaluate the development of hepatocellular carcinoma in subjects who do/do not (K8+/K8-) carry K8 variants (HR=0.8 [0.3-2.1], log-rank=0.7).

In addition, we analyzed the impact of presence of any K8 variant on the disease outcome. We did not observe any influence on the mortality (HR= 0.7 [0.6-2.7], log-rank =0.5) or HCC occurrence (log-rank=0.7, Figure 2), with calculation of power yielding low values for these analyses (0.7 and 0.5 respectively). Finally, using the Cox’s proportional hazards model, the presence of keratin variants was not associated with HCC occurrence in multivariate analysis. The multivariate model selected three independent features associated with the development of HCC in this cohort, namely older age, male gender, and high Child-Pugh score.

## Discussion

In the presented study, we analyzed the importance of K8 variants in a cohort of French patients with alcoholic liver cirrhosis. K8 R341H variant was the most common variant and was found in 8 subjects (variant frequency 3.1%), which lies well below the frequencies seen in US Caucasians with end-stage liver disease or acute liver failure (5.1 and 7.2%, respectively, Table 3) [6,19]. On the other hand, similar variant frequencies were observed in US control subjects as well as in a German cohort of patients with chronic hepatitis C (Table 3) [18,19]. Similarly, the frequency of K8 G62C variant found in our cohort is comparable to their occurrence in a large cohort of German control subjects (1.9 vs 1.6-1.8%, Table 3) [20,21]. Although the frequency of keratin variants in French population remains unknown, these data strongly suggest that K8 variants are not overrepresented in our cohort of patients with alcoholic liver cirrhosis.

**Table 3:** Frequency of K8 variants observed in previously published studies

**Patients cohort**	**Disease/ controls**		**#K8 G62C/ # subjects (%)**	**#K8 R341H/ # subjects (%)**	**Reference**
France	ALC	261	5 (1,9)	8 (3,1)	This study
Europe	Controls	4167	65 (1,6)	-	[13]
Germany	CHC	278	5 (1,8)	8 (2,9)	[18]
US	End-stage liver disease	274	4 (1,5)	14 (5,1)	[19]
	Controls	268	1 (0,373)	8 (3,0)	
Germany	ALD	215	4 (1,9)	-	[21]
	Controls	679	12 (1,8)		
Germany	Controls	560	9 (1,6)	-	[20]
Italy	PBC	201	4 (2,0)	8 (4,0)	[5]
	Controls	200	0	3 (1,5)	
US	ALF	252	4 (1,6)	18 (7,2)	[6]

*ALC*- Alcoholic Liver Cirrhosis, *CHC*-Chronic Hepatitis C, *ALD*- Alcoholic Liver Disease, *PBC*- Primary Biliary Cirrhosis, *ALF*-Acute Liver Failure.

Next, we focused on the role of K8 variants in disease outcome. Although keratins represent established tumor markers [12,22], the analyzed K8 variants did not affect the development of HCC. On the other hand, the presence of K8 R341H, but not K8 G62C or total K8 variants predisposed to non-HCC-related liver mortality. Several human association studies support the biological importance of this variant. For example, K8 R341H was significantly overrepresented in US patients with end-stage liver disease versus the control subjects [19] and in patients with acute liver failure, the presence of K8 R341H predisposed to adverse disease outcome [6].

While the higher rate of death in patients carrying K8 R341H variant is intriguing, a more powerful Kaplan-Meier analysis, which takes into account not only the number of events occurring during follow-up but also their rapidity of onset and their respective weight in the population, did not confirm these findings. Although this finding should be interpreted cautiously due to the low number of keratin variants in this cohort, the present data suggest that K8 R341H variant may not affect the outcome of alcoholic liver disease. This is supported by the fact that although keratins are well established hepatoprotective proteins, they do not offer protection in all stress situations. For example, amino-acid-altering keratin variants do not enhance TNF*a*-related liver toxicity [4] and do not predispose to progression of liver disease in patients with hemochromatosis [15,23].

## Conclusions

Our study analyzed the impact of keratin variants on disease outcome in patients with alcohol-related liver cirrhosis. Although K8 R341H variant may predispose to development of end-stage liver failure, the limited numbers of patients available as well as the low frequency of keratin variants within the cohort make it impossible for this study to conclusively clarify the significance of keratin variants in subjects with alcohol-related liver cirrhosis. Furthermore, these findings must be validated in other populations, even though the cohort used herein is well studied and was used in the past to replicate several established genetic associations [24,25]. Up to date, all reports (including ours) focusing on genetic predisposition to life-threatening events in cirrhotic patients are prone to interpretation bias related to low power analyses, a limitation that will not be overcome until coordinated work of international research consortia allows the establishment of large cohorts of patients.

## Abbreviations

K: Keratin; HCC: Hepatocellular carcinoma; ALD: Alcoholic liver disease; AFP: A-fetoprotein; DHPLC: Denaturing high-performance liquid chromatography.

## Competing interests

The authors declare that they have no competing interests.

## Authors’ contributions

VU, PN, MB and PS designed the study and wrote the manuscript. VU and ML performed the experiments. MZ, PN, PR, AS and MB collected the samples and the corresponding clinical data. PN performed the statistical analysis and PS obtained the funding. All authors read and approved the final manuscript.

## Author’s information

Valentyn Usachov Author to whom reprint requests should be addressed.

## Pre-publication history

The pre-publication history for this paper can be accessed here:

http://www.biomedcentral.com/1471-230X/12/147/prepub
